# Risk factors for malignancy in pediatric subacute/chronic focal craniocervical lymphadenopathy

**DOI:** 10.3389/fped.2024.1466116

**Published:** 2025-01-30

**Authors:** Yishai Haimi-Cohen, Eyal Elron, Lital Oz-Alcalay, Lama Hejly, Roy Hod, Liat Ashkenazi-Hoffnung

**Affiliations:** ^1^ Department for Day Hospitalization, Schneider Children’s Medical Center of Israel, Petah Tikva, Israel; ^2^ Infectious Diseases Unit, Schneider Children’s Medical Center of Israel, Petah Tikva, Israel; ^3^ Faculty of Medical and Health Sciences, Tel Aviv University, Tel Aviv, Israel; ^4^ Department of Neonatology, Schneider Children’s Medical Center of Israel, Petah Tikva, Israel; ^5^ Department of Pediatrics A, Schneider Children’s Medical Center of Israel, Petah Tikva, Israel; ^6^ Department of Otorhinolaryngology, Schneider Children’s Medical Center of Israel, Petah Tikva, Israel

**Keywords:** biopsy, benign, malignancy, cervical lymphadenopathy, lymphoma

## Abstract

**Aims:**

To describe the factors associated with malignancy in otherwise healthy children with focal persistent isolated craniocervical lymphadenopathy at low risk for malignancy, in order to aid in decisions of nodal surgical excision.

**Material and methods:**

Demographic and clinical data were retrospectively obtained for children with subacute and chronic craniocervical lymphadenopathy, treated from January 2008 to December 2020 at a general pediatric ambulatory clinic of a tertiary center. Univariate and multivariate analyses of risk factors for malignancy were performed.

**Results:**

Of the 450 children included, median age 4.2 years (interquartile age: 2.4–8.7), 25 (5.6%) were eventually diagnosed with a malignancy. In univariate analysis, factors associated with malignancy included older age, increased nodal size, location (supraclavicular and lateral cervical), systemic signs such as decreased appetite and weight loss, and abnormal imaging studies. Referral by an ear, nose, throat specialist vs. a family physician or a pediatrician was also associated with malignancy. Fever, night sweats, pruritus, hepatosplenomegaly and laboratory workup were not associated with malignancy. Twenty percent of the children older than 12.5 years and 12% of those with a lymph node diameter >31 mm were diagnosed with malignancy. Multivariate analysis showed associations with malignancy of older age and larger lymph nodes; the respective odds ratios were 1.649 (95%CI: 1.197–2.349, *p* = 0.004) for every 3 years and 2.080 (95%CI: 1.292–3.330, *p* = 0.002) for every one centimeter.

**Conclusions:**

Older age and increased nodal size can help identify children with focal craniocervical lymphadenopathy who are at increased risk for malignancy and for whom surgical intervention should be strongly considered.

## Introduction

Palpable craniocervical lymph nodes are a common finding in pediatric patients. These nodes are generally considered enlarged if measured more than 10 mm in their longest diameter (1). Enlarged nodes, i.e., lymphadenopathy, are arbitrarily categorized as acute, sub-acute and chronic, if persisting for <2, 2–6 and >6 weeks, respectively (2).

Lymphadenopathy may result from various etiologies, most of them infectious and inflammatory; malignancy is the culprit in a minority of cases. Although malignancy can be diagnosed by cytology of samples obtained using fine needle aspiration, the sensitivity of cytology in children is inadequate (3). Therefore, a definite diagnosis is based on a nodal biopsy. The decision to biopsy an enlarged lymph node is fairly easy in patients with systemic manifestations, and no evidence of infectious or inflammatory etiology. However, recommending this intervention in an otherwise healthy child with no abnormal blood tests or imaging studies is challenging given the potential surgical complications (nerve insults, infections, facial scar, the risks of a general anasthesia), costs of hospitalization and the high rates of futile pathology results that do not affect management (3–9). Finding pre-operative clinical tools that may assist physicians in the dilemma of whether to recommend surgical intervention can be of great benefit. The aim of the present study was to identify predictive factors for malignancy among generally healthy pediatric patients with focal subacute and chronic head and neck lymphadenopathy.

## Methods

### Study design and data collection

This retrospective study was conducted at a general pediatrics ambulatory clinic at Schneider Children's Medical Center, from January 2008 to December 2020. Included were generally healthy children who were referred for evaluation of sub-acute and chronic craniocervical lymphadenopathy. Ethics approval was received from our local institutional review board (RMC-19-0712). The study inclusion criteria were: (a) age <18 years; (b) enlarged focal craniocervical lymph-nodes defined as >10 mm in the cervical, submandibular/digastric and pre-auricular foci; or >5 mm in the supraclavicular foci; (c) Subacute or chronic lymphadenopathy, defined as lymphadenopathy lasting 2–6 weeks or more than six weeks, respectively. Patients with the following conditions were excluded: (a) a severe background illness, including congenital or acquired immunodeficiency or an autoimmune disease; (b) a previous history of malignancy; (c) the presence of a congenital craniocervical mass.

Data were collected by searching the electronic medical patients’ files for demographic, background and clinical variables. Background data included exposures to pets or to unpasteurized milk, a history of dental treatment, and contact with a person suspected to have tuberculosis. Clinical data included the duration from presentation to referral for evaluation, the time to the last follow-up, and physical examination findings. The latter included lymph node characteristics [location, side, size, nodal quality (tenderness, firm soft, mobile/fixed)], fever and hepatosplenomegaly. Laboratory results in the first 2 weeks after admission were also recorded, including hematology; chemistry; inflammatory markers and serology for *Epstein-Barr Virus*, *Cytomegalovirus*, *Bartonella henselea.* and *Toxoplasma gondii*; microbiology and pathological results. Reports of imaging studies were also accessed, specifically chest x-rays, and cervical and abdominal sonography.

In our institution, a general attending pediatrician is the case manager of enlarged lymph nodes’ evaluations. A joint consultation with an ear, nose, throat (ENT) specialist and a hematology-oncology specialist is made once a question regarding nodal excision arises. The decision for excision is based on the integration of clinical, laboratory and imaging data on an individual basis. As malignancies may be detected after discharge, we searched pathology reports for neoplasia in an electronic database that is common to all health insurance providers in Israel. Children who did not undergo a surgical procedure were managed with close follow-up until clinical resolution. Abnormal local sonography was defined as abnormal if one of the following parameters was present: loss of oval hypoechoic structure, loss of the hyperechoic hilum, a thickened cortex and increased disorganized vascularity (10). Malignancy was defined based on pathological reports.

### Statistical analysis

Data were reported as means ± standard deviations (SD), or as medians and interquartile ranges (IQR), as appropriate. Demographics, clinical, laboratory and imagining data were compared between children who were diagnosed with malignancy and children with benign conditions. Categorical variables were analysed using ×^2^ test. Continuous variables were compared by the *t*-test, the Mann–Whitney or the one-way ANOVA test. Multivariate logistic regression was used to assess associations between patient characteristics (independent variable), selected on the basis of the univariate analysis, and malignancy (dependent variable). The Wald test was used for calculating confidence intervals (CIs). The results are presented based on the full data set. A *p* value of ≤0.05 was deemed statistically signiﬁcant. Statistical analysis was performed using IBM SPSSv27 for Windows (SPSS Inc., Armonk, NY).

## Results

### Patient characteristics

During the study period, 450 children, (290 males, 64%) who were evaluated for sub-acute or chronic focal cervical lymphadenopathy met the inclusion criteria. The median age was 4.2 years (IQR: 2.4–8.7). Table 1 shows the demographic, clinical, laboratory and imaging characteristics of the patients, according to a diagnosis of a malignancy. Most (89%) children were referred for evaluation by their primary physicians. Empirical antibiotics were administered to 203 (45%) children.

**Table 1:** Demographic, clinical and laboratory characteristics of 450 children evaluated for sub-acute and chronic focal craniocervical lymphadenopathy according to diagnosis of malignancy.

Characteristics	No malignancy *N* = 425	Malignancy *N* = 25	*P*-value
Demographic characteristics			
Age, years, median [IQR]	4.0 [2.4–7.9]	10.3 [4.7–14.8]	**<0**.**001**
Male, *n* (%)	271 (64)	19 (76)	0.21
Clinical characteristics			
Referring physician, *n* (%)			
Primary physician	382 (90)	18 (72)	**0**.**01**
ENT specialist	40 (9)	6 (24)	
Hematologist	3 (1)	1 (4)	
Time from presentation to referral, months, median [IQR]	3 [1–10]	2 [2–3]	0.107
Time from admission to the last electronic search for pathology results, median [IQR]	3.7 [2.2–5.0]	2.9 [1.8–4.8]	0.95
Lymph node characteristics^a^			
Location*, n* (%)			
Submandibular	175 (41)	6 (24)	**<0**.**001**
Anterior cervical	72 (17)	5 (20)	
Posterior cervical	81 (19)	0	
Lateral cervical	60 (14)	8 (32)	
Supra-clavicular	11 (3)	4 (16)	
Pre-auricular	12 (3)	1 (4)	
Post auricular	11 (3)	0	
Lower cervical	2 (<1)	1 (4)	
Submental	1 (<1)	0	
Side, right/left *n* (%)	35 (32)/37 (34)	4 (57)/2 (29)	0.345
Size: maximal diameter on physical examination, cm, mean (SD)	18.4 (11.3)	28.3 (16.1)	**0**.**001**
Quality			
Soft, *n* (%)	350 (90.2)	16 (76.2)	0.187
Firm, *n* (%)	34 (8.8)	5 (23.8)	**0**.**038**
Tender, *n* (%)	86 (20)	4 (16)	0.577
Mobile, *n* (%)	381 (97.9)	19 (100%)	0.034
Fixed, *n* (%)	8 (2.1)	0	
Symptoms and signs at presentation, *n* (%)			
Fever >37.5°C	116 (27)	1 (4)	0.14
Weight loss	30 (8)	5 (23)	**0**.**031**
Pruritus	7 (2)	1 (5)	0.592
Night sweats	18 (5)	3 (14)	0.175
Loss of appetite	38 (10)	8 (36)	**0**.**01**
Enlarged liver or spleen^b^	28 (7)	3 (12)	0.320
Laboratory results			
CRP > 0.5 mg/L, median [IQR]	0.5 [0.5–8]	0.5 [0.5–22]	0.193
ESR > 20 mm/h, median [IQR]	14 [7–28]	25 [13–44]	0.109
Abnormal CBC^c^	52 (14)	6 (25)	0.118
ALT, AST ≥ 2-folds the upper limit, *n* (%)	126 (33)	6 (26)	0.48
Imaging results			
Widened mediastinum or hilar adenopathy on chest x-ray, *n* (%)	10 (3)	7 (28)	**<0**.**001**
Abnormal cranio-cervical lymph nodes’ sonography^d^, *n* (%)	139 (41)	18 (78)	**<0**.**001**
Abnormal abdominal sonography, *n* (%)	76 (18)	5 (20)	0.78

ALT, alanine aminotransferase; AST, aspartate transferase; CBC, complete blood count; CRP, C-reactive protein; ENT, ear nose and throat; ESR, erythrocyte sedimentation rate; IQR, interquartile range; SD, standard deviation.

Bold data represent statistically significant values (*p* < 0.05).

^a^ Seven children in the non-malignant group were eventually diagnosed by imaging/pathology results to have non-lymph node conditions (see the text for the specific diagnoses).

^b^ >2 cm below the ribs’ cage.

^c^ Hemoglobin: <10.5 gr/dl, White blood cells: <4.0 × 1,000 /mm^3^ or >15 × 1,000/mm^3^, platelets: <150,000/mm^3^.

^d^ Reference number (10).

The median time from presentation to referral for evaluation was 3 months (IQR: 1–9). Patients were clinically followed by treating physicians for a median of 49 days (IQR: 1–198). The median time from lymphadenopathy presentation to the study's last electronic search for pathology reports of malignancy was 3.6 years (IQR: 2.1–4.9). A total of 53 children (12%) had excisional biopsy. The median time from the initial presentation to surgical biopsy was 13.5 days (IQR: 6–61).

**Figure 1:**
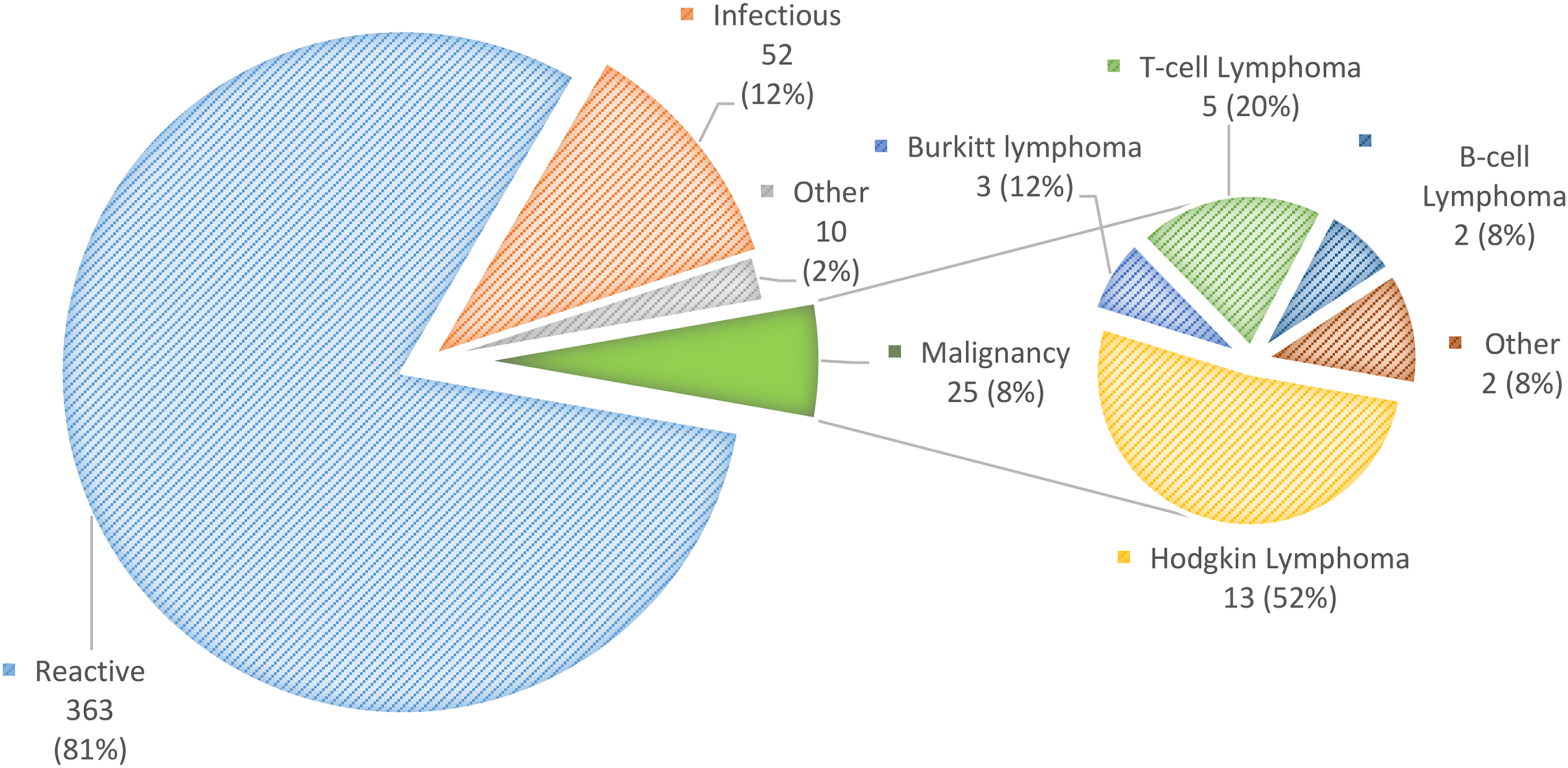
Distribution of diagnoses of 450 children with sub-acute or chronic focal craniocervical lymphadenopathy.

Twenty-five (5.6%) patients were diagnosed with malignancy according to the pathological report. The most common was Hodgkin lymphoma in 13 (52%), followed by *T*-cell lymphoma, 5 (20%); Burkitt lymphoma, 3 (12%); B-cell lymphoma, 2 (8%); nasopharyngeal carcinoma, 1 (4%); and low-grade acinic cell carcinoma, 1 (4%) (Figure 1).

Among the 425 (95%) patients with non-malignant nodal enlargement, the following diagnoses were detected: reactive lymphadenopathy, 363 (85%); infectious, 52 (12%); Kikuchi-Fujimoto disease, 2 (0.5%); and Rosai-Dorfman disease, one (0.2%). Other diagnoses were thyroglossal cysts (3), pilomatrixoma (2), myofibroma (1) and parotid pleomorphic adenoma (1).

### Characteristics compared between children with and without malignancy

**Figure 2:**
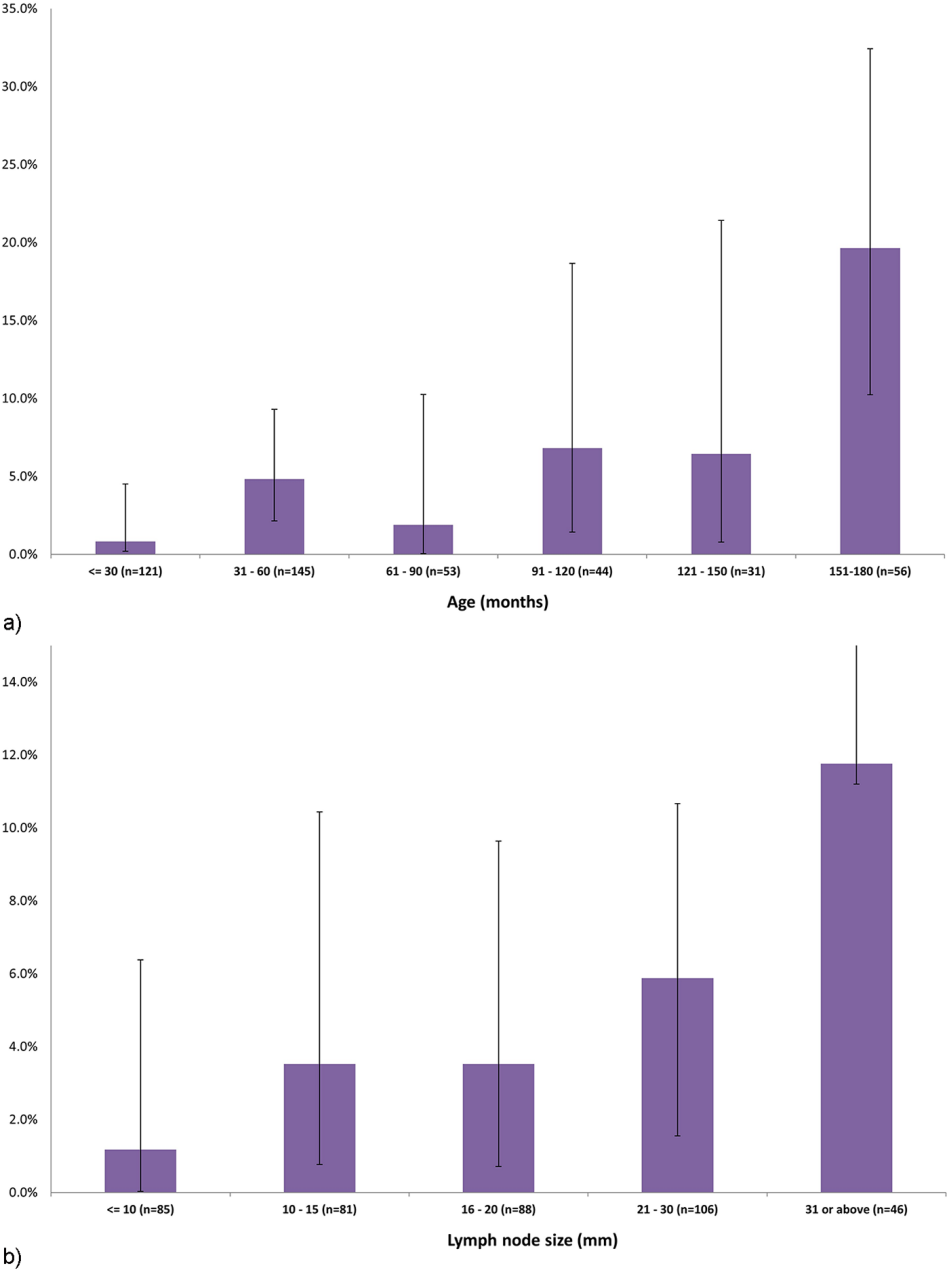
**(a)** The proportions and 95% confidence intervals of diagnosed malignancies among 450 children with sub-acute or chronic focal craniocervical lymphadenopathy, by age. **(b)** The proportions and 95% confidence intervals of diagnosed malignancies among 450 children with sub-acute or chronic focal craniocervical lymphadenopathy, by lymph node size.

In univariate analysis, among children who were diagnosed with malignancy compared to those who were not, the median age was older (10.3 years, IQR 4.7–14.8 vs. 4.0 years, IQR 2.4–7.9, *p* < 0.001) and the mean (SD) maximal nodal diameter was higher (28.3 + 16.1 cm vs. 18.4 + 11.3 cm, *p* = 0.001). Accordingly, the malignancy rate was 19.6% (95%CI: 9.5–12.8) among children aged 12.6 years or older, and 0.8% (95%CI: 0.6–3.7) among children aged 2.5 years or younger (Figure 2a). The malignancy rate was 11.8% (95%CI: 0.6–25.3) for lymph node diameters >31 mm, and 1.2% (95%CI: 1.1–5.2) for diameters <10 mm (Figure 2b). Among patients with malignancy, the nodal location was more common in the lateral, cervical and supraclavicular lymph nodes than in the submandibular region (Table 1).

Referral by an ENT specialist, compared to a family physician or pediatrician, was also more common among children diagnosed with malignancy. Systemic signs, such as a loss of appetite and weight loss, were more prevalent among children with malignancy; yet rates of fever, night sweats, pruritus, and enlarged liver or spleen did not differ between the groups (Table 1).

Laboratory results including C-reactive protein (CRP) values, erythrocyte sedimentation rate, complete blood count and hepatocellular liver enzymes did not differ statistically between children with and without malignancy. Abnormal imaging studies, including abnormal chest x-ray and abnormal findings on targeted cervical sonography, were more common among children with than without malignancy (Table 1).

### Multivariate logistic regression analysis for factors associated with malignancy

Multivariate analysis identified two factors significantly associated with malignancy (Table 2). The odds ratio (OR) for every 3-year increase in age was 1.649 (95%CI: 1.197–2.349, *p* = 0.004) and the OR for each centimeter increase in lymph node size was 2.080 (95%CI: 1.292–3.330, *p* = 0.002).

**Table 2:** Multivariate logistic regression for predictive factors for malignancy in children with sub-acute or chronic focal craniocervical lymphadenopathy.

Characteristic	OR	95%CI	*P* value
Age, for every 3 years	1.649	1.197–2.349	**0** **.** **004**
Lymph node diameter, for every increase in one centimeter	2.080	1.292–3.330	**0**.**002**
Loss of appetite	3.128	0.744–13.160	0.120

A total of 450 children were included in the analysis. Bold data represent statistically significant values (*p* < 0.05), OR, odds ratio.

## Discussion

In this study of 450 children who were referred for evaluation of sub-acute or chronic focal craniocervical lymphadenopathy, multivariate analysis identified older age and larger nodal size as two factors predicting malignancy. The respective ORs were 1.649 (95%CI: 1.197–2.349, *p* = 0.004) for every increase in 3 years in age and 2.080 (95% CI: 1.292–3.330, *p* = 0.002) for every increase in nodal size of one centimeter. Moreover, we managed to quantify the risks for malignancy of specific age groups and nodal sizes. Accordingly, 20% of the patients older than 12.5 years and 12% of those with a lymph node diameter greater than 31 mm were diagnosed with malignancy.

The two factors identified herein were previously reported as associated with malignancy among children (5, 11–18). Soldes et al. identified an association between increasing age and the probability of malignancy (11), and others reported ORs of 1.072 (95%CI: 1.001–1.148) and 1.209 (95%CI: 1.082–1.350) for every one-year increase in age (12, 13). In a study that included children and adults, the mean age was significantly higher in the malignant than the non-malignant group (6). The increased incidence of lymphadenopathy in young children, which is probably due to a higher rate of pharyngeal infections in early childhood, can partially explain the lower rate of malignancy in younger than older children. Interestingly, a recent study from Korea found that malignancy was more common in younger than older children (5).

The results are inconsistent regarding the lymph node diameter cut-off values that would best imply likelihood of malignancy. However, most studies suggest that a nodal size above 2–3 cm is associated with an increased risk for malignancy. One study found that a cut-off of 2.6 cm yielded a sensitivity of 76.9% and a specificity of 67.4%, for malignancy, with a negative predictive value of 90.6%, and a positive predictive value of 41.7% (5). Others suggested that a diameter ≥2 cm yielded a higher likelihood of malignancy (15). Several pediatric studies reported statistically significant higher rates of a malignant disease compared to a benign condition in nodes with diameters >3 cm: 69.5% vs. 33.3%, 58.3% vs. 19% and 85.6% vs. 33.2% (16–18). Accordingly, a higher mean nodal diameter was reported in excised nodes with malignant compared to benign pathology results, 3.51 ± 2.78 vs. 2.61 ± 0.91 cm (12). In contrast, in a study of 98 children who underwent surgical excision of persistent enlarged lymph nodes, an association was not found between node size and a diagnosis of malignancy (19).

The rate of malignancy in our patients was substantially lower than previously reported: 5.5% vs. 23%–72% (5, 11, 16–18, 20). This disparity may be related to our cohort consisting of generally healthy children with sub-acute or chronic focal craniocervical lymphadenopathy as the only presumed risk factor for malignancy. In contrast, most other studies included diversified populations with characteristics that could affect the risk for malignancy. These included patients with a history of a neoplasia (11, 14), adults (14), patients with generalized lymphadenopathy (11, 13–18, 20) and children with acute presentation (17, 18, 21). Furthermore, some studies comprised only patients with post-nodal excisions, who were apparently a-priori at higher risk of malignancy (5, 11–14, 20). It is plausible that the presentation of relatively minor general manifestations among our patients with malignancy reflects successful containment of the proliferative process within the presented nodes. Indeed, in a recent study of asymptomatic pediatric patients with persistent cervical lymphadenopathy, the rate of malignancy was low, only 1.5% (4).

Similar to our study, Bozlac et al. looked for risk factors of malignancy among patients with cervical lymphadenopathy in a general pediatric clinic. In their prospective study, six patients were diagnosed with malignancy compared to 212 children with other conditions (21). This yielded a low rate of 2.8% of malignancy. In accordance with our results, the children with malignancies tended to be older than those without. However, unlike our findings, the association was not statistically significant, possibly due to the small number of patients with neoplasia. While 58.7% of their patients had acute symptoms, all our patients were evaluated for sub-acute or chronic lymphadenopathy. Another methodological difference is our longer follow-up period, 3.6 years (IQR: 2.1–4.9) vs. 8 weeks, which is crucial to avoid missing late diagnoses of malignancy.

The associations with malignancy found in our univariate analysis corroborate several other studies, specifically for parameters like weight loss (18, 22) supra-clavicular location (13, 14, 16–18, 22, 23), abnormal chest x-ray (13, 16–18) and targeted cervical sonography (4, 14, 20, 21). The diagnostic advantage of the latter has been supported by two findings. First, the maximal nodal width-to-length ratio aided in predicting malignancy, with an OR of 52.08 (95%CI: 16.10–168.6) (20). Second, the absence of a nodal fatty hilum was shown to be useful in deciding on surgical biopsy for suspected malignancy (4). However, Ingolfsdottir et al. reported an 80% false positive and 15% false negative result of pre-operative sonographic findings, compared to histology results of malignant and benign conditions, respectively (23). Others did not find statistically significant differences between sonographic results of patients with benign and malignant lymph nodes (22). These results underscore the caution needed in implying nodal sonographic interpretation in clinical decisions of lymphadenopathy evaluation.

In accordance with others, we did not find systemic symptoms, namely night sweats and fever, to be associated with malignancy (12, 22). We also did not find utility of erythrocyte sedimentation rate, CRP, complete blood count and hepatomegaly in predicting malignancy, thus corroborating other studies (6, 11, 13, 14, 16–18, 21). These results may be due to differences in tumor types and subtypes, and in the degree of their local and remote proliferation.

The shared decision-making model has been shown to decrease the number of biopsies in patients with lymphadenopathy (4, 6, 9). Employing this model by sharing in-house ENT and hematology-oncology physicians in the decision to recommend a biopsy probably contributed to the relatively low rate of invasive interventions in our cohort. It is plausible that this practice also contributed to parental confidence in the suggested management approach.

The strengths of our study are its large scale, the focus on a homogenous cohort with a specific condition, and the practical findings, which may be useful for clinicians evaluating pediatric patients with focal craniocervical lymphadenopathy. However, our study has several limitations. First is the retrospective design. Nevertheless, data were collected extensively from standardized electronic files, and included a wide range of demographic, clinical and laboratory variables, as well as imaging investigations. A second limitation is that some diagnoses of malignancy may have been missed, as not all the patients underwent a surgical biopsy and some may have been diagnosed after the study ended. However, this is unlikely due to the long duration of the follow-up i.e., the median time from the lymphadenopathy presentation to the last electronic search was 3.6 years (IQR: 2.1–4.9). Thirdly, our results represent a single-center experience of an ambulatory pediatric setting. Therefore, prospective studies of children with focal craniocervical lymphadenopathy are needed to validate our results, preferably with standardized indications for nodal biopsy.

In conclusion, our findings suggest that age and nodal size can contribute to clinicians’ decisions regarding excision of an enlarged focal craniocervical lymph node in a generally healthy child. A lymph node >2 cm in school-aged children or adolescents, or >3 cm in toddlers should be strongly considered for excision.

## Data Availability

The original contributions presented in the study are included in the article/Supplementary Material, further inquiries can be directed to the corresponding author.
